# Enhancing empathy and attitudes toward dementia among formal caregivers through virtual reality: a randomized controlled trial

**DOI:** 10.1093/geroni/igaf145

**Published:** 2025-12-15

**Authors:** Dorothy Bai, Huei Ling Chiu, Shu-Cheng Lin, Kelvin Tan Cheng Kian, Yeh-Liang Hsu, Gong-Hong Lin

**Affiliations:** School of Gerontology and Long-Term Care, College of Nursing, Taipei Medical University, Taipei City, Taiwan; School of Gerontology and Long-Term Care, College of Nursing, Taipei Medical University, Taipei City, Taiwan; School of Gerontology and Long-Term Care, College of Nursing, Taipei Medical University, Taipei City, Taiwan; Minor in Applied Ageing Studies Programme, S R Nathan School of Human Development, Singapore University of Social Sciences, Singapore; Gerontechnology Research Center, Yuan Ze University, Taoyuan City, Taiwan; International PhD Program in Gerontology and Long-Term Care, College of Nursing, Taipei Medical University, Taipei City, Taiwan

**Keywords:** Immersive simulation, Perspective-taking, Professional training, Long-term care

## Abstract

**Background and Objectives:**

Enhancing empathy and attitudes among formal caregivers is essential for improving dementia care quality. However, traditional educational approaches often lack the emotional depth needed to foster person-centered care. The primary objective was to evaluate whether an immersive virtual reality (VR) intervention produced greater improvements than a traditional lecture-based education program in empathy and dementia attitudes; secondary outcomes were caregiver burden and psychological distress.

**Research Design and Methods:**

This parallel-group randomized controlled trial involved 160 formal caregivers randomly assigned to an immersive VR intervention or a time-matched lecture-based comparator. The intervention was delivered as a single 3-hr session comprising three consecutive VR segments, each followed by guided small-group reflection, with brief breaks between segments. Outcomes were measured at baseline, post-intervention, and at 1 month. Analyses followed the intention-to-treat principle using generalized estimating equations.

**Results:**

The VR intervention group exhibited greater improvements in empathy (2.21, 95% CI: 0.42–4.01, *p* = .016) and attitudes toward dementia (1.18, 95% CI: 0.18–2.17, *p* = .021) immediately post-intervention compared to the control group. However, these effects were not sustained at the 1-month follow-up.

**Discussion and Implications:**

A single-session, multi-segment VR program can produce immediate improvements in empathy and attitudes toward dementia compared with lecture-based education. As effects were not maintained at 1 month, implementation may pair modular VR with low-burden refreshers to support durability while maintaining feasibility. Future research should compare delivery schedules and assess longer-term and behavioral outcomes.

**Clinical Trial Registration:**

NCT06072274.

Innovation and Translational Significance Traditional lecture-based education often falls short of improving empathy and attitudes essential for person-centered dementia care. We implemented a randomized controlled trial comparing a single-session, multi-segment immersive virtual reality (VR) program with guided small-group reflection to a time-matched lecture and observed immediate improvements in empathy and attitudes. To help these gains endure beyond 1 month, programs may pair modular VR with brief refreshers. Health systems and educators may integrate VR modules into in-service training, and policymakers may incorporate standardized scenarios and outcome metrics into workforce standards to support wider implementation and improve care for older adults.

## Background and objectives

Dementia is a progressive neurological disorder characterized by significant cognitive impairment, affecting millions worldwide (GBD 2019 Dementia Forecasting Collaborators, 2022). As the global population ages, its prevalence is expected to rise substantially, creating major challenges for healthcare systems and caregivers. Formal caregivers frequently struggle to perceive and respond to the needs of people with dementia, increasing stress and reducing care quality (Holst & Skär, 2017; Miyamoto et al., 2010; Sampaio et al., 2021). Deficits in empathy can exacerbate these challenges by limiting sensitivity to care recipients’ needs and undermining person-centered support (Åström et al., 1991). Such shortcomings may heighten caregiver stress and compromise care outcomes (Gillis et al., 2023). Consequently, enhancing caregiver empathy and fostering positive attitudes toward individuals with dementia are essential for delivering compassionate and effective care (Dokos et al., 2023; Zhi et al., 2024).

The importance of empathy and attitudes can be further understood through theoretical frameworks. The person-centered care model emphasizes that empathetic understanding and respectful attitudes are central to preserving dignity, reducing behavioral symptoms, and improving quality of life for individuals with dementia (Kitwood, 1997). Empathy theory further highlights cognitive perspective-taking and affective resonance as mechanisms that enhance communication and reduce caregiver stress (Davis, 1994; Hojat et al., 2018). Together, these frameworks underscore why strengthening empathy and attitudes is vital in dementia care and provide the rationale for evaluating innovative approaches such as VR education.

Traditional dementia education typically relies on lectures and workshops (Surr & Gates, 2017). While these approaches provide foundational knowledge, they are often passive and non-interactive, limiting learners’ emotional engagement and their ability to apply knowledge in real-world caregiving situations. Moreover, they rarely convey the lived experiences of people with dementia, restricting caregivers’ understanding and responsiveness (Zhi et al., 2024). These shortcomings highlight the need for innovative educational interventions to enhance empathy and reshape caregiver attitudes. Virtual reality (VR) has emerged as a promising tool for experiential learning within healthcare education (Pottle, 2019; Wijma et al., 2018; Zhi et al., 2024). By immersing caregivers in simulated environments that approximate the lived experiences of individuals with dementia, VR can facilitate firsthand insights into the daily struggles associated with the condition (Zhi et al., 2024). Such immersive experiences hold significant potential to promote deeper empathy and foster more positive attitudes toward care recipients.

However, previous randomized or controlled studies examining VR-based education for dementia care have yielded inconsistent findings (Stargatt et al., 2021; Sung et al., 2022). For example, a 3-month program that combined monthly 1-hr immersive sessions with peer support reported immediate improvements in empathy and attitudes that were sustained at 1 month (Sung et al., 2022). In contrast, a single-session workshop comparing VR with non-VR education did not show overall advantages for VR on primary outcomes, although subgroup analyses indicated greater empathy gains among older participants in the VR condition (Stargatt et al., 2021).

These mixed results likely reflect heterogeneity in several design features and contexts, including the degree of immersion and interactivity, whether guided reflection or peer discussion was included, the cultural and linguistic fit of materials, the underlying educational theory, participant characteristics such as age and language background, and variability in outcome measures (Stargatt et al., 2021; Sung et al., 2022; Zhi et al., 2024). Collectively, prior evidence suggests that pairing immersive exposure with structured opportunities for meaning-making and tailoring content to the caregiving context may be important for achieving reliable effects (Pottle, 2019; Wijma et al., 2018; Zhi et al., 2024).

Building on these insights, we adopted a single-session VR format to balance feasibility with educational benefits given formal caregivers’ limited availability. To retain repeated exposure within one visit, the session comprised three brief segments, each followed by a guided small-group discussion; content addressed common dementia-related situations across home settings, public transport, and other public places, and community care centers. This design was informed by empathy theory and experiential learning, in which concrete experience followed by reflective observation supports perspective-taking and attitudinal change (Davis, 1994; Kolb, 2014). Intervention procedures are described in the Methods section. The primary objective was to evaluate whether this structured VR program was more effective than lecture-based education in improving empathy and attitudes toward dementia among formal caregivers; secondary outcomes were caregiver burden and psychological distress.

## Research design and methods

This study employed a parallel randomized controlled trial design to evaluate the effectiveness of an immersive VR intervention on enhancing empathy and shifting attitudes toward dementia among formal caregivers. The trial was conducted and reported in accordance with the Consolidated Standards of Reporting Trials (CONSORT) guidelines and was registered with ClinicalTrials.gov (identifier: NCT06072274). The authors assert that all procedures contributing to this work comply with the ethical standards of the relevant national and institutional committees on human experimentation and with the Helsinki Declaration of 1975, as revised in 2013. All procedures involving human subjects were approved by Taipei Medical University Joint Institutional Review Board (approval no.: N202308052). Written informed consent was collected from all participants.

## Participants

The study was conducted in community settings in Taipei, Taiwan. Participants were recruited through digital flyers, emails, and targeted invitations shared via LINE, a widely used communication app among caregiving professionals in Taiwan. Recruitment was conducted in collaboration with community-based service organizations, including adult day care centers, home-based care providers, and local care management units. Eligible participants were primarily care attendants and home care workers. Inclusion criteria were (1) age ≥ 20 years, (2) proficiency in Chinese, and (3) current employment as a formal caregiver providing care for individuals with dementia. We set the age threshold at ≥20 years to ensure independent consent capacity, consistent with Taiwan’s legal age of majority when the protocol was prepared. Exclusion criteria included (1) inability to use a VR device due to physical or mental conditions, (2) aversion to or discomfort with VR devices, and (3) history of severe motion/VR sickness.

The required sample size was estimated using G*Power 3.1 (Faul et al., 2009) with an alpha level of 0.05, statistical power of 0.90, and a medium effect size (Cohen’s *d* = 0.5), based on prior research involving VR-based dementia education for formal caregivers (Sung et al., 2022). The calculation indicated that a minimum of 128 participants would be sufficient to detect statistically significant effects on the primary outcomes. To account for a potential attrition rate of 25%, the final target sample size was increased to 160 participants (80 per group).

## Randomization, allocation concealment, and blinding

Participants were randomly assigned through block randomization using computer-generated sequences to either an experimental group receiving the VR intervention or a control group receiving traditional lecture-based education. An independent researcher, uninvolved in participant assessments, created the allocation sequence with a block size of four and ensured allocation concealment by placing assignments in sealed envelopes. Participants were then allocated to the intervention or control group in a 1:1 ratio within each block. Due to the nature of the intervention, blinding of participants was not possible. However, researchers responsible for outcome assessments and data analysis remained blinded to the allocation throughout the study.

## Intervention

The VR intervention was designed to provide an immersive, first-person simulation of the lived experiences of people with dementia, aiming to enhance caregivers’ empathy and attitudes. The program incorporated key principles from experiential learning to support emotional engagement and perspective-taking (Kolb, 2014). It consisted of three short VR segments delivered consecutively within a single 3-hr session. Based on pilot testing, recommending a single-session schedule due to limited availability and staffing constraints among formal caregivers, we adopted this design to balance feasibility with educational benefits and maximize completion. Each segment was followed by a guided small-group discussion, with brief breaks between segments.

VR content was delivered using Samsung Gear VR head-mounted displays with spatial audio in 360-degree video format. Each segment included approximately 5 min for setup and safety checks, a 10–15-min first-person VR segment, and about 30 min of guided reflection and discussion in small groups of five to eight participants, with 5–10-min breaks between segments. Scenarios were fixed (not user-selectable) to ensure consistent exposure. Facilitators with healthcare backgrounds and dementia education training followed a semi-structured guide with fixed core prompts: (1) immediate emotional responses to the scenario; (2) moments that were challenging for the person with dementia and why; (3) implications for communication and environmental adjustments in care; and (4) one concrete action to try in practice.

The three VR segments depicted common scenarios experienced by individuals with dementia in home settings, on public transportation, and in other public places, and in community care centers, including forgetting where to get off a train, difficulty recognizing familiar places or caregivers, misinterpreting safety cues, and visual hallucinations such as insects in food or briefly appearing people. The content was originally developed by Silver Wood Co., Ltd (Japan), then translated into Mandarin and culturally adapted in collaboration with a Taiwan-based organization. To ensure contextual and educational relevance, the adapted materials were reviewed by dementia care professionals, gerontological nursing faculty, and experienced formal caregivers during pilot work, which informed refinements in language, pacing, and usability. The localized content was used under license from the original developer.

Participants assigned to the control group received a time-matched, 3-hr didactic program covering fundamental dementia knowledge, symptom recognition, and caregiving strategies. Content was delivered by dementia-education staff with healthcare backgrounds using slides and short videos; no VR or simulation elements were included. Sessions used a fixed syllabus and standardized materials to ensure consistency across groups.

## Outcome measures

Data were collected using standardized questionnaires administered at three time points: baseline (T_0_), post-intervention (T_1_), and at 1-month follow-up (T_2_). The primary outcome measures were empathy and attitudes toward dementia, respectively assessed using the Jefferson Scale of Empathy (JSE; ­Gamvrouli et al., 2024; Hojat et al., 2018) and the Approaches to Dementia Questionnaire (ADQ; Lintern et al., 2000; Lintern, 2001). The JSE is a 20-item scale on a seven-point Likert format (with higher scores indicating greater empathy), and the ADQ is a 19-item five-point Likert scale (with higher scores reflecting more positive attitudes). Secondary outcomes included caregiver burden, assessed by the Zarit Burden Interview (ZBI; Cejalvo et al., 2024; Yap, 2010), and mental health status, measured by the Brief Symptom Rating Scale (BSRS; Chen et al., 2005; Sanjaya et al., 2023). The ZBI (22 items on a five-point Likert scale, with higher scores indicating a greater burden) and the BSRS (five items on a five-point Likert scale, with higher scores reflecting more-severe symptoms) were administered only at the baseline and at the 1-month follow-up, as immediate changes post-intervention were not anticipated.

## Statistical analysis

Baseline characteristics were summarized using means, standard deviations, and proportions, and between-group balance was evaluated using standardized mean differences. For JSE, ADQ, ZBI, and BSRS scores, between-group differences in mean changes from baseline to post-test and 1-month follow-up were examined using generalized estimating equations (GEEs) that included group, time, and group-by-time interaction terms. Each GEE model assumed a Gaussian distribution with an identity link, an exchangeable working-correlation structure, and robust (sandwich) standard errors. The coefficient for the group × time interaction represents the difference-in-differences (DiD) effect, comparing the change in the intervention group relative to the control group. Effect sizes (Cohen’s *d*) were calculated by dividing the between-group mean difference in change scores by the pooled baseline standard deviation. The post-intervention (T1) assessment was prespecified as the primary time point. To account for multiple comparisons across the two primary outcomes (JSE and ADQ) at T1, *p*-values were adjusted using the Benjamini–Hochberg procedure, controlling the false discovery rate at .05. All analyses followed the intention-to-treat principle and were conducted using IBM SPSS Statistics, Version 28 (IBM Corp., Armonk, NY, USA). A nominal significance level of 0.05 was used.

## Results

**Figure 1.**
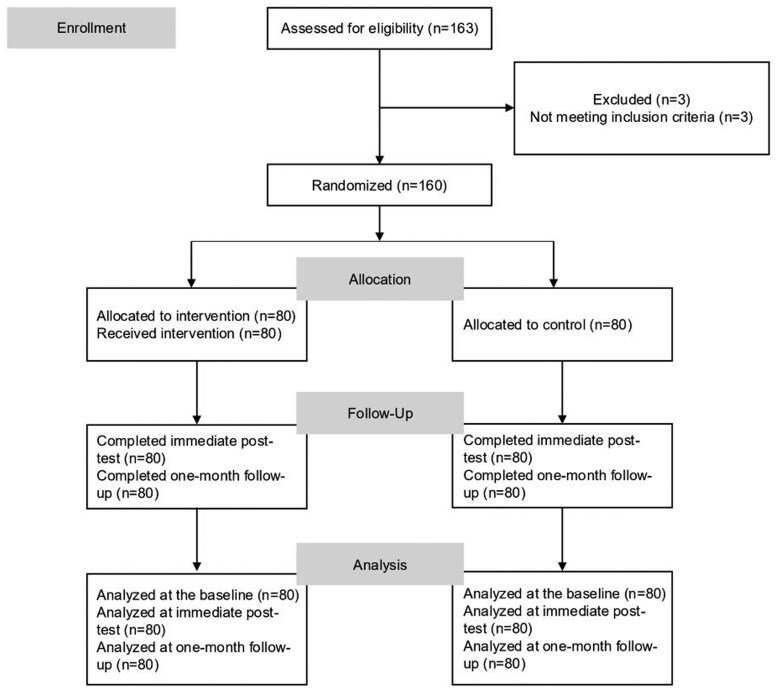
Flow chart of the study.

The study included 160 participants who were randomly assigned to either the control group (*n* = 80) or the intervention group (*n* = 80). All randomized participants completed the full intervention and were included in the final analysis. No participants dropped out or were excluded following group allocation. Participant flow is illustrated in Figure 1. Demographic and baseline characteristics are summarized in Table 1. The average age was 49.24 years. Most participants were female (86.9%) and held a university degree (70.4%). The table also lists baseline scores for empathy (JSE), attitudes toward dementia (ADQ), caregiver burden (ZBI), and psychological distress (BSRS).

**Table 1.** Characteristics of participants (*N *= 160).

Characteristics	Total	Control *n *= 80	Intervention *n *= 80	SMD
**Age (years), mean±SD**	49.24 ± 10.84	48.39 ± 10.43	50.09 ± 11.24	0.16
**Gender, *n* (%)**				0.26
** Male**	21 (13.1%)	7 (8.8%)	14 (17.5%)	
** Female**	139 (86.9%)	73 (91.2%)	66 (82.5%)	
**Educational level, *n* (%)**				0.25
** Junior high school**	10 (6.3%)	5 (6.3%)	5 (6.3%)	
** Senior high school**	37 (23.3%)	23 (29.1%)	14 (17.4%)	
** University or higher**	112 (70.4%)	51 (64.6%)	61 (76.3%)	
**Marital status, *n* (%)**				0.09
** Married**	36 (45.0%)	31 (46.3%)	67 (41.9%)	
** Single (never married)**	29 (36.3%)	34 (54.0%)	63 (39.4%)	
** Divorced or widowed**	15 (18.7%)	15 (18.7%)	30 (18.7%)	
**Years of dementia care, mean ± *SD***	3.67 ± 4.01	3.45 ± 3.42	3.88 ± 4.54	0.10
**Days of dementia care per week, mean ± *SD***	3.91 ± 1.82	3.93 ± 1.93	3.90 ± 1.70	0.02
**Received dementia training, *n* (%)**				0.17
** Yes**	148 (92.5%)	76 (95.0%)	72 (90.0%)	
** No**	12 (7.5%)	4 (5.0%)	8 (10.0%)	
**Work-related stress, mean ± *SD***	4.60 ± 2.04	4.34 ± 2.15	4.86 ± 1.91	0.25
**Family/friend with dementia, *n* (%)**				0.03
** Yes**	85 (53.1%)	43 (53.8%)	42 (52.5%)	
** No**	75 (46.9%)	37 (46.2%)	38 (47.5%)	
**Baseline measurement, mean ± *SD***				
** JSE scores**	116.98 ± 13.41	116.64 ± 14.15	117.33 ± 12.71	0.05
** ADQ scores**	70.74 ± 6.90	70.55 ± 7.13	70.93 ± 6.71	0.05
** ZBI scores**	36.58 ± 14.43	36.73 ± 14.69	36.44 ± 14.26	0.02
** BSRS scores**	5.07 ± 3.96	4.85 ± 3.83	5.29 ± 4.09	0.11

*Note*. ADQ = Approaches to Dementia Questionnaire; BSRS = Brief Symptom Rating Scale; JSE = Jefferson Scale of Empathy; *SD* = standard deviation; SMD = Standardized mean difference; ZBI = Zarit Burden Interview.

We compared differences in JSE, ADQ, ZBI, and BSRS scores between the control and intervention groups (Table 2). For empathy (JSE), the GEE model showed a significant between-group difference in change at post-intervention (coefficient = 2.21, 95% CI: 0.42–4.01, *p* = .016; Cohen’s *d* = 0.16, 95% CI: 0.03–0.30). For dementia attitudes (ADQ), the between-group difference in change was also significant at post-intervention (coefficient = 1.18, 95% CI: 0.18–2.17, *p* = .021; Cohen’s *d* = 0.17, 95% CI: 0.03–0.31). These between-group effects remained significant after Benjamini–Hochberg adjustment (JSE, adjusted *p* = .021; ADQ, adjusted *p* = .021). At the 1-month follow-up, between-group differences for JSE and ADQ were not significant. For secondary outcomes, caregiver burden (ZBI) and psychological distress (BSRS) did not differ significantly between groups at 1 month.

**Table 2.** Effects of the virtual reality intervention on outcome measures: analysis using generalized estimating equations.

Outcome variable	Interaction coefficient (group × time) (95% CI)	*SE*	*p*
***Primary outcome***			
**JSE scores**			
** Post-intervention (T_1_ vs T_0_)**	2.21 (0.42, 4.01)	0.92	**.016**
** 1-month follow-up (T_2_ vs T_0_)**	1.34 (−5.04, 7.72)	3.25	.681
**ADQ scores**			
** Post-intervention (T_1_ vs T_0_)**	1.18 (0.18, 2.17)	0.51	**.021**
** 1-month follow-up (T_2_ vs T_0_)**	1.14 (−1.73, 4.00)	1.46	.436
***Secondary outcomes***			
**ZBI scores**			
** 1-month follow-up (T_2_ vs T_0_)**	1.04 (−5.12, 7.19)	3.14	.741
**BSRS scores**			
** 1-month follow-up (T_2_ vs T_0_)**	−0.25 (−1.71, 1.66)	0.86	.977

*Note*. ADQ = Approaches to Dementia Questionnaire; BSRS = Brief Symptom Rating Scale; JSE = Jefferson Scale of Empathy; *SE* = standard error; ZBI = Zarit Burden Interview; T_0_ = baseline; T_1_ = post-intervention; T_2_ = 1-month follow-up. For the two primary outcomes at the primary time point (T1), *p*-values were adjusted using the Benjamini–Hochberg method (JSE, adjusted *p* = .021; ADQ, adjusted *p* = .021).

## Discussion

This randomized controlled trial evaluated the effectiveness of an immersive virtual reality educational program in enhancing empathy and improving attitudes toward dementia among formal caregivers. The findings indicated that the VR intervention led to immediate improvements in empathy and attitudes compared to traditional lecture-based education. However, these effects were not sustained at the 1-month follow-up, highlighting potential limitations in the long-term impacts of a short-term VR intervention.

The improvement in empathy scores immediately post-intervention aligns with prior research, which demonstrated that immersive, first-person experiences can promote deeper empathetic understanding by allowing individuals to “walk in another’s shoes” (Sung et al., 2022; Zhi et al., 2024). The VR intervention was particularly effective in enhancing formal caregivers’ ability to recognize non-verbal cues, understand patients’ lived experiences, and adopt a more person-centered approach to dementia care. These aspects are critical for improving the quality of care provided to individuals with dementia and reinforcing the role of empathy in caregiving.

Similarly, attitudes toward dementia improved immediately following the VR intervention, suggesting that immersive experiences can challenge preexisting misconceptions and promote more positive and respectful perspectives toward individuals with dementia (Sung et al., 2022). The first-person perspective of the VR program facilitated experiential learning, reinforcing the importance of recognizing the capabilities and dignity of persons with dementia. These findings support the growing body of literature advocating for VR as an effective tool in healthcare education to enhance attitudinal change (Sung et al., 2022; Zhi et al., 2024).

Regarding secondary outcomes, we observed no significant differences between the groups in caregiver burden, as measured by the ZBI, or mental health status, as measured by the BSRS, at the 1-month follow-up. This may indicate that while the VR intervention can improve empathy and attitudes in the short term, it might be insufficient on its own to address broader contributors to caregiver well-being, such as patient aggression or reduced independence in activities of daily living, contributing to caregiver burden and mental health problems (Gillis et al., 2023; Miyamoto et al., 2010; Parrotta et al., 2020). Future research should explore the integration of VR education with additional support strategies, such as peer discussions, counseling, or skill-building workshops, to achieve more comprehensive benefits for caregivers’ well-being.

Although empathy and attitude scores improved immediately, effect sizes at post-test were small, which may reflect limited room for improvement given relatively elevated baseline empathy and attitudes among formal caregivers in this study. Future studies may consider targeting caregivers with lower baseline empathy or attitudes, or incorporating screening to identify subgroups who may benefit most from VR-based interventions. In addition, the effects were not sustained at the 1-month follow-up. This pattern is consistent with the nature of affective learning, in which emotional insight and perspective-taking tend to fade without reinforcement (Wood & Rünger, 2016). Empathy theory suggests that practice and feedback are needed to convert short-lived empathic arousal into durable attitudes and behaviors (Davis, 1994; Hojat et al., 2018). In experiential learning, repeated cycles of concrete experience followed by guided reflection promote consolidation (Kolb, 2014). The single-session schedule, selected for feasibility and reach, likely limited opportunities for ongoing practice and reinforcement. Studies using longer or multi-session VR programs have reported more durable effects (Sung et al., 2022). From a practical training perspective, a single-session format maximizes feasibility and reach, but benefits may attenuate without reinforcement. Future implementations should evaluate reinforcement strategies, such as brief booster sessions and workplace reflection prompts, or multi-session schedules that balance feasibility with maintenance, and assess durability as well as downstream behavioral and care-quality outcomes.

A growing literature on VR interventions for informal caregivers shows a similar pattern of immediate gains in empathy and, in some programs, attitudes, with variable maintenance across studies (Huang et al., 2024; Wijma et al., 2018). Several studies also report reductions in caregiver burden at follow-up, whereas effects on psychological distress are often small or non-significant (Han et al., 2020; Huang et al., 2024; Karana & Paun, 2023). Informal caregivers’ continuous contact and emotional closeness to the person with dementia provide frequent opportunities to apply perspective-taking in daily routines (Nemcikova et al., 2023). In contrast, formal caregivers work within shift schedules, role boundaries, and standardized procedures that may limit practice and reinforcement, which could attenuate durability or shift effects toward attitudes rather than burden (Stargatt et al., 2021; Sung et al., 2022; Zhi et al., 2024). These contextual differences may help explain the variability in maintenance observed across populations.

There are limitations to this study. First, the sample was restricted to formal caregivers in a single urban area, which may limit the generalizability of findings to other regions or to informal caregivers. Selection bias is also possible, as those who opted to participate may have differed from nonparticipants in motivation, availability, or openness to VR-based learning. Second, the use of self-reported measures introduces the risk of social desirability bias. Caregivers may have reported more empathetic attitudes or behaviors aligned with socially acceptable norms rather than their true perceptions. To mitigate this risk, all questionnaires were completed anonymously, and participants were assured their responses would be used solely for research purposes without implications for their professional standing. Nonetheless, this potential bias should be considered when interpreting the results.

This randomized controlled trial shows that immersive, first-person VR can improve empathy and attitudes among formal caregivers in the short term. As the demand for dementia care continues to grow, scalable and experiential educational tools such as VR may offer a promising complement to existing caregiver training strategies. However, the lack of sustained effects suggests a need for training models that extend engagement beyond a single session while preserving feasibility for formal caregivers. For implementation, brief, scenario-based segments delivered in small groups with guided reflection and discussion are recommended. Programs may embed brief booster sessions and workplace reflection prompts to support retention or, when feasible, distribute content across multiple sessions. Future research should examine reinforcement strategies, delivery schedules, and longer-term outcomes such as caregiver behavior and care quality, and explore whether similar approaches are effective for informal caregivers and across both community and institutional care settings.

## Data Availability

Data are available from the corresponding author on reasonable request. The study was preregistered at ClinicalTrials.gov (NCT06072274).
